# The Capabilities of Multimodal Magnetic Resonance Morphometry to Diagnose Complex Cases of Focal Cortical Dysplasia (Pilot Study)

**DOI:** 10.17691/stm2026.18.4.03

**Published:** 2026-08-31

**Authors:** A.M. Shevchenko, N.Y. Alsahanova, E.L. Pogosbekyan, A.I. Batalov, N.E. Elagina, A.A. Kopaneva, A.V. Bernstein, M.G. Sharaev, I.N. Pronin

**Affiliations:** MD, PhD, Junior Researcher; Radiologist, Department of Radiologic and Radioisotope Diagnostic Methods; N.N. Burdenko National Medical Research Center of Neurosurgery, Ministry of Health of the Russian Federation, 16 4th Tverskaya-Yamskaya St., Moscow, 125047, Russia; PhD Student; Research Engineer, BIMAI-Lab; Skolkovo Institute of Science and Technologies, 30, Bldg. 1, Bolshoy Boulevard, Moscow, 121205, Russia; PhD, Medical Physicist; Researcher, Department of Radiologic and Radioisotope Diagnostic Methods; N.N. Burdenko National Medical Research Center of Neurosurgery, Ministry of Health of the Russian Federation, 16 4th Tverskaya-Yamskaya St., Moscow, 125047, Russia; MD, PhD, Senior Researcher; Radiologist, Department of Radiologic and Radioisotope Diagnostic Methods; N.N. Burdenko National Medical Research Center of Neurosurgery, Ministry of Health of the Russian Federation, 16 4th Tverskaya-Yamskaya St., Moscow, 125047, Russia; Radiologist, Department of Radiologic and Radioisotope Diagnostic Methods; N.N. Burdenko National Medical Research Center of Neurosurgery, Ministry of Health of the Russian Federation, 16 4th Tverskaya-Yamskaya St., Moscow, 125047, Russia; Radiologist, Department of Radiologic and Radioisotope Diagnostic Methods; N.N. Burdenko National Medical Research Center of Neurosurgery, Ministry of Health of the Russian Federation, 16 4th Tverskaya-Yamskaya St., Moscow, 125047, Russia; DSc, Professor, BIMAI-Lab; Skolkovo Institute of Science and Technologies, 30, Bldg. 1, Bolshoy Boulevard, Moscow, 121205, Russia; DSc, Senior Lecturer; Head of BIMAI-Lab; Skolkovo Institute of Science and Technologies, 30, Bldg. 1, Bolshoy Boulevard, Moscow, 121205, Russia; MD, DSc, Professor, Academician of the Russian Academy of Sciences, Head of the Department of Radiologic and Radioisotope Diagnostic Methods; N.N. Burdenko National Medical Research Center of Neurosurgery, Ministry of Health of the Russian Federation, 16 4th Tverskaya-Yamskaya St., Moscow, 125047, Russia

**Keywords:** focal cortical dysplasia, MR morphometry, artificial intelligence, machine learning, pharmacoresistant epilepsy, multimodal MRI

## Abstract

Focal cortical dysplasia (FCD) is a leading cause of pharmacoresistant epilepsy. Up to 30–50% of cases remain MRI-negative on routine visual assessment. MR morphometry improves dysplasia detection; however, conventional approaches are limited to the analysis of T1-weighted images and only two features (gray–white matter blurring and cortical thickness abnormalities).

**The aim of the study** was to compare the diagnostic efficiency of the conventional T1-based two-feature algorithm and a multimodal multi-feature algorithm incorporating machine learning.

**Materials and Methods:**

The study included four patients with histologically verified FCD (2 patients with type I and 2 patients with type IIB according to the Blümcke classification). All patients underwent MRI using the HARNESS protocol. MR morphometry was performed using two independent algorithms.

The conventional method included FreeSurfer-based assessment of 2 features (the analysis of cortical thickening and gray–white matter boundary blurring) on T1-weighted images.

The multimodal morphometric algorithm incorporated T1/T2-weighted images and FLAIR, and included the following features: gray–white matter transition abnormalities on T1, T2, and T2-FLAIR images (Blurring_T1/T2/FLAIR); detection of local T2- and FLAIR-hyperintense signal abnormalities (CR_T2/FLAIR); assessment of FLAIR intensity distribution heterogeneity to identify blurred boundaries or the transmantle sign (Variance); measurement of signal irregularity on FLAIR images to detect the regions of increased contrast (Entropy). Machine learning models integrating all extracted features were used to generate final FCD prediction maps (MLP and XGBoost).

**Results:**

In two cases, where FCD had typical T1-signs (cortical thickening or well-defined demarcation impairment), both algorithms demonstrated equal diagnostic performance. In the third case, the classical approach failed to detect pathology, since only T1-weighted images were analyzed, whereas the multimodal approach enabled to identify dysplastic areas (only T2/FLAIR images showed the changes). In the fourth case, the conventional algorithm detected a single lesion, while the multimodal algorithm identified an additional second lesion, which influenced the surgical treatment strategy.

**Conclusion:**

Multimodal MR morphometry with artificial intelligence outperforms the conventional approach in complex cases (MRI-negative and multifocal lesions) and should be considered an important supplementary technique in preoperative planning.

## Introduction

Focal cortical dysplasia (FCD) is one of the most common causes of drug-resistant epilepsy, especially in children, and is a leading cause of structural epilepsy in candidates for surgical treatment [1–3]. The only treatment for such patients is radical surgical resection of the epileptogenic focus; however, the success of the surgery directly depends on the accurate preoperative determination of the localization of the boundaries of the dysplastic cortical area [1].

The gold standard for FCD diagnosis is visual assessment using high-resolution magnetic resonance imaging (MRI) according to specialized protocols, such as HARNESS. However, this approach has significant limitations. Dysplastic lesions are often very small, located deep within complex sulci and gyri, or show only subtle structural changes [3]. As a result, a substantial proportion of FCD cases (reported to be as high as 30–50%) remain undetected by routine visual analysis, contributing to what is known as MRI-negative epilepsy [1, 4]. Additionally, interpreting MRI results is subject to considerable inter-expert variability and requires significant time investment [3]. Because of these challenges, there is a strong need for objective and sensitive analytical methods capable of detecting hidden structural abnormalities. MRI morphometry based on voxel image analysis and data post-processing, offers a way to quantitatively assess key features of FCD, such as local cortical thickening and blurring of the gray–white matter boundary [1, 4].

Our studies have shown the use of MR morphometry to enable to increase the detection rate of FCD type I focal cortical dysplasia by 18% (up to 85%) compared to standard visual assessment (66%), which is of particular importance due to the challenges in diagnosing this category of dysplasia [1]. MR morphometry has been clinically proven to be valuable in identifying lesions in patients with prolonged MRI-negative epilepsy. Subsequent histological studies confirmed the presence of dysplasia. Postoperatively, these patients experienced sustained remission of epileptic seizures [4].

Currently, efforts are focused on improving the method specificity and reducing the number of false-positive results. Promising directions include integrating MR morphometry with quantitative MRI techniques, such as MR fingerprinting, as well as the development of machine learning algorithms to automatically distinguish true pathological lesions from normal variants [2, 3]. Moreover, the studies are underway to assess how the quality of original MR images impacts post-processing outcomes — the aspect is critical for the proper standardization of the method [2].

In summary, exploring the diagnostic potential of MR morphometry, optimizing its algorithms, and integrating it into clinical practice are highly relevant goals to enhance patient selection for surgical treatment and improving its overall effectiveness.

**The aim of the study** was to compare the diagnostic efficiency of two MR morphology algorithms in patients with pharmacoresistant epilepsy suspicious for focal cortical dysplasia.

The first algorithm (N.N. Burdenko National Medical Research Center of Neurosurgery) is a classical method based solely on the analysis of T1-weighted images, evaluating two features (cortical thickening and gray–white matter blurring).

The second algorithm (BIMAI-Lab, Skolkovo Institute of Science and Technologies) is a multimodal method (T1, T2, FLAIR) with the calculation of an expanded set of morphometric features.

## Materials and Methods

The prospective study included four patients diagnosed with FCD types I and II, all suffering from pharmacoresistant structural epilepsy (one patient with obvious FCD; one MRI-negative patient; one with FCD visible only on T2 and T2-FLAIR imaging; and one with extensive FCD). Prior to surgery, all patients underwent brain MRI following the HARNESS protocol using either a General Electric Signa HDxt (GE Healthcare, USA) 3.0 Tesla scanner with an 8-channel head coil or a MAGNETOM Skyra (Siemens, Germany) 3.0 Tesla scanner with a 32-channel head coil. The MRI images were analyzed by a multidisciplinary team consisting of radiologists, epileptologists, and neurosurgeons. The imaging findings, clinical data, and other diagnostic test results were presented at a multidisciplinary conference to determine the surgical strategy and scope aimed at resecting the epileptogenic zone. After surgery, FCD was confirmed in all patients; two were diagnosed with FCD type I, and two with FCD type IIB according to Blümcke classification.

The study was conducted in compliance with the 2024 Declaration of Helsinki and approved by the Ethics Committee of the N.N. Burdenko National Medical Research Center of Neurosurgery (Russia).

### Multimodal MRI morphometry algorithm using machine learning (BIMAI-Lab, Skolkovo Institute of Science and Technologies)

Within the study framework, a set of quantitative feature maps associated with FCD were generated and normalized against a “healthy” database to improve the sensitivity to subtle changes including MRI-negative cases.

The following morphometric features were computed: Blurring_T1: a feature aimed at detecting blurred boundaries between gray and white matter measured on T1-weighted MRI;

Blurring_T2: a feature detecting blurred gray–white matter boundaries measured on T2-weighted MRI;

Blurring_FLAIR: a feature detecting blurred gray–white matter boundaries measured on FLAIR MRI;

CR_T2: a feature aimed at detecting an increased signal (the gray–white matter hyperintensity boundary) measured on T2-weighted MRI. CR (concentration rate) indicates abnormally high signal intensity in the area, and this feature typically detects hyperintensities well;

CR_FLAIR is a feature aimed at detecting an increased signal (the hyperintensity boundary between gray/white matter) measured by FLAIR MRI. CR indicates that the signal is abnormally high for the area. This feature typically detects hyperintensity well;

Variance is a feature that helps detect blurriness at the interface between gray and white matter or a transmantle sign measured by FLAIR MRI. This feature is more related to the irregular distribution of intensity in the area; i.e. blurring at the boundary or a transmantle sign in white matter leads to an increase in the value of Variance; Entropy is a feature that enables to identify the areas of increased signal (hyperintensity boundaries of gray matter/white matter) and a transmantle sign characterized by higher contrast between the area of interest and its surroundings. This feature is calculated using FLAIR and reflects the randomness of the signal;

MLP, XGBoost are the models built on features (they generalize the information from the features and provide predictions).

The output involved the formation of maps detailing morphometric and contrast features as well as prediction maps from the trained machine learning models (MLP and XGBoost [5]) [6]. The main stages of this algorithm are described below.

#### Data preparation and spatial normalization

The original 3D MRI images (T1, T2, FLAIR) were anonymized and converted to the NIfTI format. The T1 images were registered to the symmetrical standard MNI152 template space (based on 152 MRIs) [7]. T2 and FLAIR modalities were then transformed into MNI152 space using linear registration to ensure voxel-wise correspondence across the modalities. After registration, intensity normalization (z-score) and correction for MRI signal inhomogeneity were performed. Tissue segmentation and the creation of probabilistic maps of gray matter (GM), white matter (WM), and cerebrospinal fluid were conducted using SPM12 [8]; voxel values on each map represented the probability of belonging to the corresponding tissue type. Brain masks were generated using HD-BET [9].

#### Extraction of morphometric features from T1

Morphometric maps of cortical thickness, curvature, and sulcal depth were calculated from T1 images using FreeSurfer (recon-all) [10]. To refine cortical boundaries, the FLAIR modality was used as an auxiliary input. The resulting surface maps were converted into volumetric maps and registered to the MNI152 template space for subsequent voxel-wise analysis.

#### Boundary blurring maps between gray and white matter for T1, T2, and FLAIR

For each modality, a “gray–white matter” boundary map was created based on individual thresholds derived from intensity distributions within gray matter (GM) and white matter (WM). For T1, the thresholds were defined as:

Tlow=μGM+δ⋅σGM;

Thigh=μWM−δ⋅σWM,

where μ and σ represent the mean and standard deviation of intensities within the corresponding tissue types, and δ is an empirical parameter adjusted to satisfy the condition *T_low_*<*T_high_* (δ=0.4).

For T2 and FLAIR, a similar approach was applied, accounting for the inverse GM/WM contrast relative to T1:

Tlow=μWM+δ⋅σWM;

Thigh=μGM−δ⋅σGM,

where δ=0.03, δ*=*0.04 for T2 and FLAIR, respectively.

Then a binary map was generated: a voxel was assigned the value “1” if its intensity fell between *T_low_* and *T_high_*, and “0” otherwise.

To eliminate gaps and merge adjacent voxels with value “1” into connected clusters, a 3D convolution was performed using a kernel of ones sized 5×5×5, resulting in the final feature map formed for each modality.

#### Contrast and textural-statistical features

Let *X*(*v*) denote the image intensity at voxel *v*. For each voxel, we consider a local cubic neighborhood *W_m_*(*v*)*=*{*t*:*|t–v|∞≤m*}, where *m* is the half-width of the window (the radius of the voxel neighborhood), and |·|_∞_ represents the Chebyshev norm. The window size is therefore (2*m*+1)·(2*m*+1)·(2*m*+1) voxels, yielding *|W_m_*(*v*)*|*=(2*m*+1)^3^.

To identify local regions with elevated signals, concentration maps of bright values (CR) were created based on T2 and FLAIR images. For each voxel *v*, the neighborhood *W_m_*(*v*) (*m*=4) was considered, and the voxel intensities within the region were sorted in descending order:

X(i)(v)≥⋯≥X(|Wm(v)|)(v),

where *X*_(_*_i_*_)_(*v*) is the intensity value in voxel, |*Wm*(*v*)|=9^3^.

The CR feature was defined as the sum of a subset of the highest intensity values, excluding the brightest outliers:

CR(v)=∑i=k+1k+gX(i)(v),

where *g* (*g*=105) is the number of bright voxels considered, and *k* (*k*=15) is the number of top maximum values excluded to suppress isolated outliers.

Using FLAIR data, local entropy as a texture feature was additionally computed based on the gray-level co-occurrence matrix (GLCM) within the local area *W_m_*(*v*) (*m*=5). Rényi entropy (Entropy) of order α (α=2) was used:

Hα(v)=11−αlog∑i,jfi,j(v)α,

where *f_i,j_*(*v*)^α^ are the normalized GLCM elements.

Additionally, local variance (Variance) was calculated from the FLAIR images as:

V(v)=1|Wm(v)|−1∑t∈Wm(v)(X(t)−Xmean(v))2,

where $X_{mean}(v)=\frac{1}{\left|W_{m}(v)\right|}\sum_{t\in W_{m}(v)}X(t)$,

where *m*=4.

#### Normalization relative to the control group and postprocessing

The baseline was established using MRI data from healthy hemispheres of patients with FCD (n=176). For each feature, the upper and lower quantiles, *q_l_*=0.975 and *q_s_*=0.025, respectively, were computed from this sample. Then, the feature map values *X*(*v*) in each voxel *v* were transformed into the deviation maps *X*(*v*)*_n_* according to the following rule:

X(v)n=X(v)−ql, if X(v)>ql;

X(v)n=0, if qs<X(v)<ql;

X(v)n=qs−X(v), if X(v)<qs.

Thus, nonzero values on the normalized map indicate the degree of deviation from the norm.

To suppress noise, all connected regions were automatically identified, and those with volumes smaller than 50 voxels were discarded.

#### Predictions of machine learning models

Based on the set of feature maps, additional prediction maps were generated using multilayer perceptron (MLP) and XGBoost models. The models were trained on a sample of 176 patients utilizing original MRI images in MNI space, probabilistic maps of gray and white matter, and feature maps (blurring for T1, T2, and FLAIR; CR for T2 and FLAIR; Rényi entropy; local variance), as well as cortical morphometric measurements obtained from FreeSurfer (cortical thickness, curvature, and sulcal depth). Each model output is a voxel-wise probability map indicating the likelihood of that voxel belonging to a FCD lesion. These prediction maps were further post-processed by extracting connected components and filtering out small clusters (smaller than 50 voxels).

#### Inverse transformation of the findings into individual space

To ensure accurate clinical visualization, all feature maps (blurring, CR, Entropy, Variance) and the model prediction maps (MLP, XGBoost) originally computed in template space, were transformed into the patient native T1-weighted MRI space using the inverse transformations obtained during spatial normalization. As a result, a comprehensive set of voxel-wise maps was generated for each patient in their individual anatomical space:

Blurring for T1, T2, and FLAIR showing voxel-wise parametric maps that highlight regions where there is pronounced blurring of the gray–white matter boundary compared to normal, across different MRI modalities;
CR for T2 and FLAIR displaying local concentrations of hyperintense signals, reflecting the areas of abnormal intensity increases typical of altered tissue in FCD regions; Entropy for FLAIR illustrating textural heterogeneity, it helps identify zones where signal regularity is disrupted relative to normal tissue;
Variance for FLAIR capturing local intensity variability, indicating abnormal signal fluctuations that may accompany structural cortical changes associated with FCD;
Probability maps of FCD presence generated by the machine learning models (MLP and XGBoost).

### Classical MR morphometry algorithm — N.N. Burdenko National Medical Research Center of Neurosurgery

T1-weighted MRI images were used. The main stages of the algorithm are described below.

#### Data preparation and spatial normalization

The original T1 MRI images were converted from DICOM format to NIfTI format. Then, using the recon-all command from FreeSurfer version 7.4.1 [11], binary masks of the cortex, white matter, and subcortical structures were generated. Normalized T1 images were also obtained; these underwent correction for magnetic field inhomogeneity and global linear intensity scaling so that the mean intensity values in the white matter across all subjects matched a standardized reference value.

#### Cortical thickness map calculation

The data obtained via recon-all included the information on cortical thickness. Using the mri_surf2vol command, the data were converted into NIfTI format.

#### Group cortical thickness statistical analysis

For each patient, voxel-wise comparisons of their cortical thickness map were made against the corresponding maps of a healthy volunteer group using the mri_glmfit command. This produced a voxel-wise parametric map indicating deviations in cortical thickness from the normative values for each patient.

#### Calculation of the gray–white matter boundary blurriness map

For each normalized T1 image, a binary mask of the gray–white matter boundary was created based on individually calculated thresholds, derived from the intensity distributions in gray matter (GM) and white matter (WM). Prior to threshold calculation, noise was removed from the T1 images using a non-local means filtering method [12]. The thresholds were defined as:

Tlow=μ+1/2⋅σGM;

Thigh=μ−1/2⋅σWM,

where μ and σ represent the mean and standard deviation of intensities in the respective tissue types. When generating the binary mask, voxels with intensities falling between *T_low_* and *T_high_* were assigned a value of “1”; otherwise, they were set to “0.” The resulting binary mask of the gray–white matter boundary underwent clustering, followed by removal of clusters that did not contact or intersect with the cortical mask. An exclusion mask was also created and subtracted from the gray–white boundary mask. This exclusion mask comprised the regions within 6 mm of the thalami and the regions within 2 mm of subcortical structures: lateral ventricles, thalami, caudate nuclei, putamen, globus pallidus, hippocampi, amygdalae, ventral parts of the diencephalon, cerebellar white matter, cerebellar cortex, brainstem, posterior part of the corpus callosum (isthmus), mid-posterior corpus callosum (splenium), central corpus callosum (body), mid-anterior corpus callosum (rostral part), and anterior corpus callosum (genu). The cortical mask was subtracted from this exclusion mask. After subtracting the exclusion mask from the gray–white boundary mask, a 3D convolution with a 5×5×5 kernel of ones was performed. The resulting image was then used for group-level statistical analyses.

#### Calculation of group statistics for the blurring of the gray–white matter boundary

For each patient, voxel-wise comparisons were conducted using the mri_ glmfit command to compare the blurring maps of the gray–white matter boundary against the corresponding maps from a group of healthy volunteers. The process yielded a voxel-wise parametric map for each patient, highlighting the areas with significantly increased blurring of the gray–white matter boundary compared to the normal reference.

## Results

MRI morphometric data from four patients were analyzed using two different algorithms.

In the series of four clinical cases involving the patients with pharmacoresistant focal epilepsy, MRI morphometry was performed using two independent methods: the classical approach (analyzing only T1-weighted images by assessing two features — cortical thickening and gray–white matter blurring) and a multimodal method (integrating T1, T2, and FLAIR images to compute over 10 features including Blurring_ T1/T2/FLAIR, CR_T2/FLAIR, Variance, Entropy, followed by a final classification using machine learning models such as MLP and XGBoost).

In the first two cases, both algorithms produced comparable results: one patient showed a dysplasia zone in the left temporo-parietal region, while another exhibited it in the left temporo-insular area; the histological analysis confirmed FCD in both. In the third case, the classical method failed to detect any structural abnormalities, whereas the multimodal algorithm successfully identified a dysplasia zone. In the fourth case, the classical method detected only one area of change, while the multimodal method revealed an additional region of structural alteration, which impacted the surgical treatment strategy.

### Clinical case 1

*Patient*: male, 50 years old, suffering from pharmacoresistant focal tonic seizures and FCD in the left temporal lobe.

**Figure 1.**
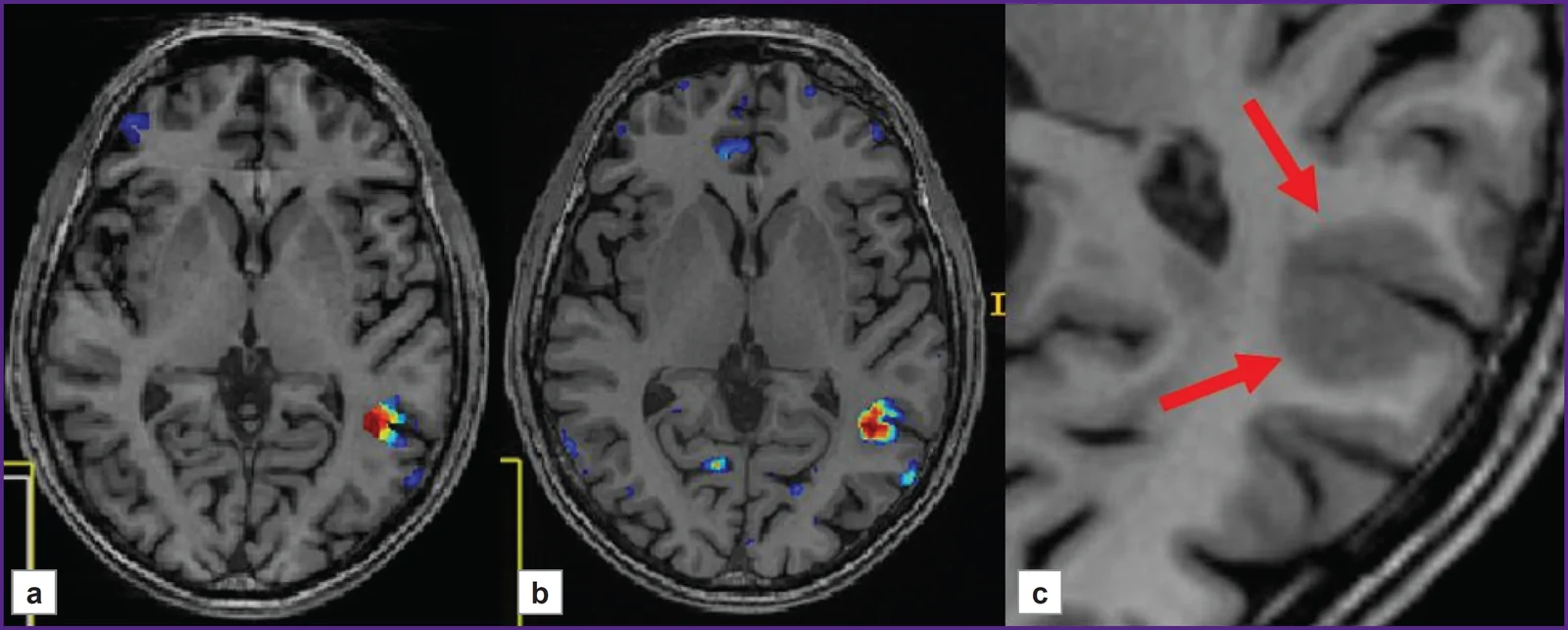
MRI scan of a 50-year-old man with focal cortical dysplasia in the left temporoparietal region: (a) classical algorithm: cortical thickening (red area); (b) multimodal algorithm: confirmation of cortical thickening (red area); (c) T1 images showing the cortical thickening in the left temporo-parietal region, with the arrows indicating the area of change

*Findings*. The classical morphometric analysis identified a localized area of cortical thickening in the left temporo-parietal region (Figure 1 (a)). The multimodal algorithm fully confirmed the presence of identical structural abnormalities in the same location with the same cortical thickening feature (Figure 1 (b)). Comparing the two methods allowed precise delineation of the cortical dysplasia zone (Figure 1 (c)). The histological examination of surgical tissue confirmed FCD type IIB. In this case, both algorithms demonstrated similar effectiveness.

### Clinical case 2

**Figure 2.**
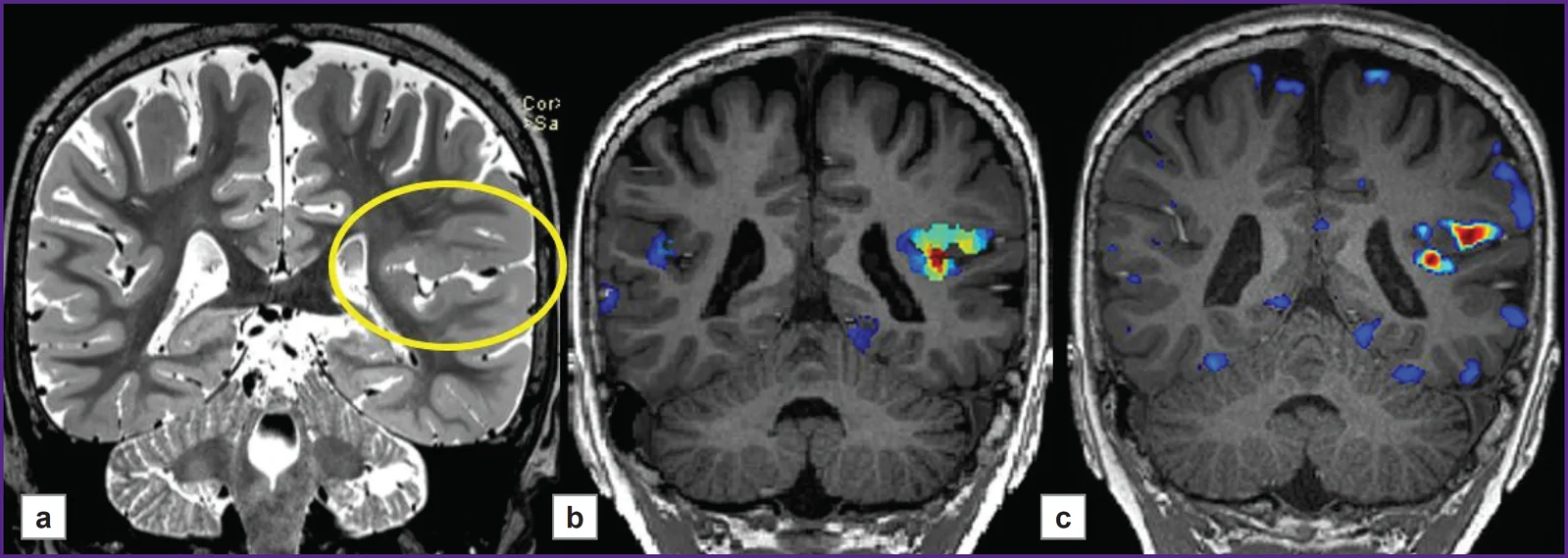
MRI of a 10-year-old boy with focal cortical dysplasia in the left temporo-insular region: (a) standard MRI: no visible abnormalities (highlighted in an oval); (b) classical morphometry: a zone of disrupted demarcation; (c) multimodal morphometry: confirmation of the affected zone

*Patient*: a 10-year-old boy, with pharmacoresistant oroalimentary automatisms and FCD in the left fronto-temporal region. The visual analysis of high-resolution MRI did not reveal any structural abnormalities (Figure 2 (a)).

*Findings*. The classical algorithm detected the gray–white matter blurring in the left temporo-insular area (Figure 2 (b)). The multimodal method confirmed the presence of the same abnormalities in the same location with similar features (Figure 2 (c)). Both methods helped refine the surgical strategy, and histological verification confirmed FCD type I. As in the first case, no significant difference in diagnostic value was observed between the two algorithms.

### Clinical case 3

**Figure 3.**
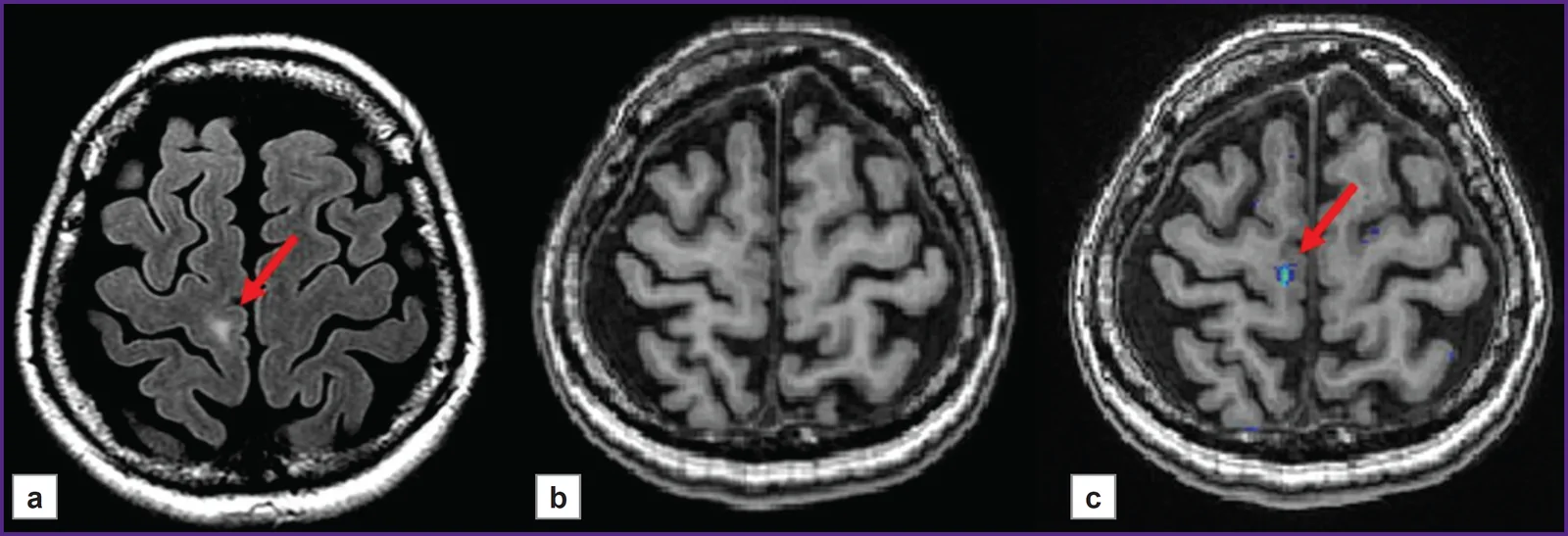
MRI of a 45-year-old woman with focal cortical dysplasia in the right precentral gyrus: (a) structural MRI (T2/FLAIR) with the arrow pointing to the lesion area; (b) classical morphometry: no abnormalities detected; (c) multimodal morphometry: clear dysplasia zone highlighted, with the arrow indicating the area of change

*Patient*: female, 45 years old, with pharmacoresistant focal tonic seizures and FCD in the right precentral gyrus. Structural MRI images showed a distinct area of gray–white matter blurring in the right precentral gyrus, visible only on T2 and FLAIR sequences (Figure 3 (a)).

*Findings*. Classical MRI morphometry (analyzing only T1 images and two features) failed to detect any structural abnormalities (Figure 3 (b)), missing the dysplasia obvious on T2/FLAIR. In contrast, the multimodal method working with T1, T2, and FLAIR and using artificial intelligence (AI) (the entirety of features with the following MLP/XGBoost classification), accurately identified the lesion in the right precentral gyrus, fully matching the structural MRI findings (Figure 3 (c)). Through the multi-feature analysis (including Blurring_T2, CR_FLAIR, Variance, Entropy) and the trained models, the multimodal approach proved sensitive even to those FCD forms, which show no cortical thickening or gray–white matter blurring on T1 but manifest clearly on T2/FLAIR.

### Clinical case 4

*Patient*: a 21-year-old woman with pharmacoresistant focal tonic seizures and extensive FCD in the right frontal lobe.

**Figure 4.**
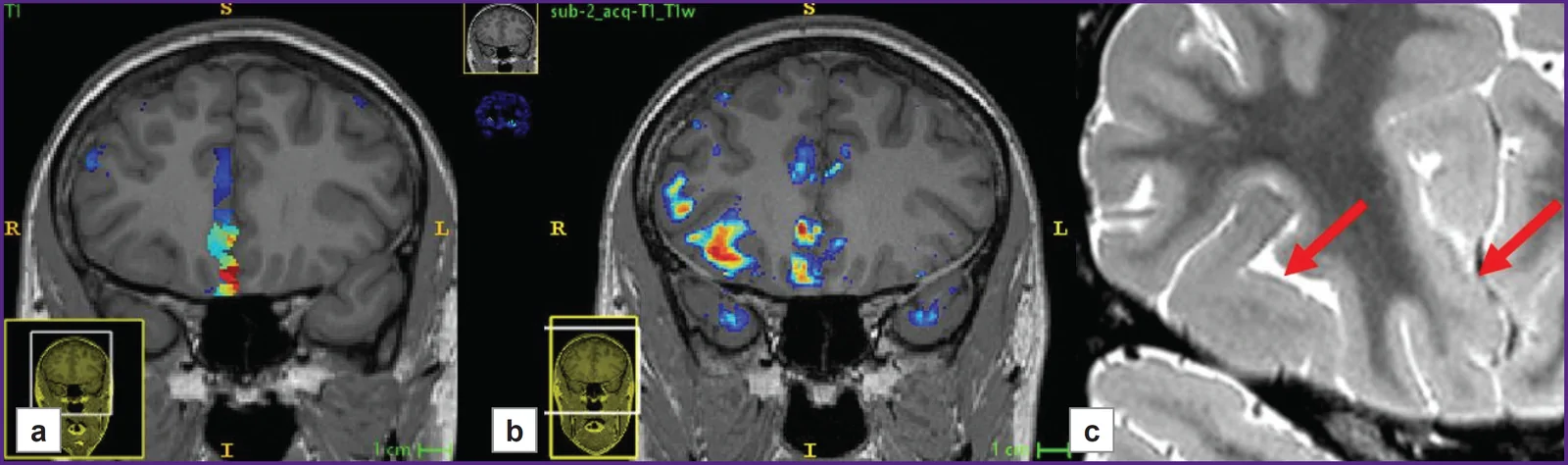
MRI of a 21-year-old woman with multifocal focal cortical dysplasia in the right frontal lobe: (a) classical morphometry: a lesion identified only in the straight gyrus; (b) multimodal morphometry: two affected areas detected (straight and inferior frontal gyri); (c) T2-weighted MRI revealing two zones of focal cortical dysplasia (straight and inferior frontal gyri), with the arrows marking the areas of alteration

*Findings*. Using the classical analysis, a zone of structural changes was visualized limited to the right straight gyrus (Figure 4 (a)). The multimodal approach not only confirmed this area but also revealed an additional region of structural abnormalities located in the right inferior frontal gyrus (Figure 4 (b)). The detailed comparison with structural MRI data identified the areas of disrupted cortico-medullary differentiation in both gyri (Figure 4 (c)). The additional lesion was detected due to the broad spectrum of morphometric features and their subsequent integration using AI models. The traditional algorithm evaluating only two features proved to be less informative regarding the second lesion. The expanded area of interest obtained through the multimodal method served as the basis for revising the surgical strategy, resulting in an increased volume of resection. The histological verification confirmed FCD type I validating the appropriateness of the extended approach.

## Discussion

Focal cortical dysplasia (FCD) is one of the most common causes of pharmacoresistant epilepsy in both children and adults, with radical surgical treatment possible only when the epileptogenic substrate is precisely localized preoperatively [13, 14]. According to the study [15], up to 30–40% of FCD cases remain “visually negative” on standard high-resolution MRI analysis. In recent years, quantitative MRI morphometry methods have been actively developed to improve diagnostic sensitivity. The best-known among these is the MAP (morphometric analysis program) algorithm, implemented in the FreeSurfer package, which evaluates cortical thickening and the gray–white matter blurring on T1-weighted images [15, 16]. This approach, similar to that used at the N.N. Burdenko National Medical Research Center of Neurosurgery, has demonstrated good sensitivity for classical FCD forms. However, the method limitations related to its monomodal nature (T1 only) and the analysis of just two features, can lead to false negatives results in atypical or multifocal lesions [17, 18].

In the presented series of clinical cases, we compared the classical T1-oriented algorithm (two features) with a multimodal, multi-feature approach integrating the machine learning models (MLP, XGBoost). In the first two cases, where FCD showed typical T1 features (cortical thickening or clear demarcation disruption), both algorithms showed comparable efficiency. It confirms that classical morphometry remains a reliable tool in routine scenarios [19].

The key differences were found in cases 3 and 4. In the third case, the FCD zone was visible only on T2 and FLAIR images, not on T1. Some histological subtypes of FCD (especially type I) are known not to show cortical thickness changes or clear demarcation on T1 but manifest as increased signal intensity on T2/FLAIR due to disrupted myelination and gliosis [20, 21]. The multimodal algorithm using the features such as blurring on T2, CR on FLAIR, Variance, and Entropy, enabled to detect these changes, whereas the T1-only algorithm yielded a false negative result. The similar advantages of multimodal morphometry have recently been demonstrated in the studies [22, 23] (the combination of T1 and T2/FLAIR with deep learning outperformed monomodal approaches).

In the fourth case, the multimodal algorithm identified an additional, previously unnoticed focus in the inferior frontal gyrus. According to literature data, multifocal FCD occur in 5–10% of cases; however, such foci are frequently missed by visual analysis and classical morphometry, particularly when lesion features differ in prominence [24]. It is precisely the broad feature set and the machine learning models ability to capture nonlinear relationships that enabled to detect the second focus. Comparable findings were reported by Spitzer et al. [25] (using XGBoost on eight morphometric features increased the multifocal FCD detection rates from 60 to 89%).

Thus, the clinical cases we present underscore the need to move from universal, limited T1-based methods toward personalized, multimodal AI solutions. The suggested approach combining the analysis of T1, T2, and FLAIR sequences, the calculation of multiple features (including blurring, CR, Variance, Entropy), and the application of machine learning models enables to reduce false negative results and detect the hidden and multifocal FCD forms. This advancement is critically important for planning radical surgical interventions.

It should be noted that the present study results are limited by a small sample size (4 patients) that precludes a comprehensive statistical analysis. Further research on larger patient cohorts is necessary to confirm the effectiveness of multimodal MRI morphometry and the informativeness of the suggested methodology.

## Conclusion

Multimodal MR morphometry using T1, T2, and FLAIR images combined with an expanded set of morphometric features and machine learning models, demonstrated higher sensitivity than the traditional T1-based algorithm in detecting focal cortical dysplasia, especially in MRI-negative and complex cases. This approach enabled a more precise assessment of the extent of structural abnormalities and improved preoperative planning, supporting the promising potential of integrating multimodal MR morphometry into clinical practice.
